# Direct Ice Packing for Voice Changes During Thyroid Radiofrequency Ablation: A Retrospective Case Series

**DOI:** 10.1155/crie/7160959

**Published:** 2026-02-17

**Authors:** Mei-Chen Yeh, Wei-Shin Yu, Hsiang-Lin Lee, Yu-Shen Lee, Kai-Lun Cheng

**Affiliations:** ^1^ Division of Metabolism and Endocrinology, Department of Internal Medicine, Chi Mei Medical Center, Tainan, Taiwan, chimei.org.tw; ^2^ School of Medicine, Chung Shan Medical University, Taichung, Taiwan, csmu.edu.tw; ^3^ Department of Internal Medicine, Division of Endocrinology and Metabolism, Chung Shan Medical University Hospital, Taichung, Taiwan, csh.org.tw; ^4^ Department of Surgery, Chung Shan Medical University Hospital, Taichung, Taiwan, csh.org.tw; ^5^ Department of Medical Imaging, Chung Shan Medical University Hospital, Taichung, Taiwan, csh.org.tw

## Abstract

Voice change is a recognized complication of radiofrequency ablation (RFA) for thyroid nodules, primarily resulting from thermal injury to the recurrent laryngeal nerve or adjacent structures. Although most cases resolve spontaneously over several weeks to months, timely intervention may help reduce patient discomfort and anxiety. This retrospective case series examined five patients who developed voice changes during or immediately after RFA for benign thyroid nodules and were managed with direct ice packing. Ipsilateral vocal cord (VC) hypomobility was confirmed via real‐time laryngeal ultrasound, followed by immediate application of ice to the skin overlying the thyroid. VC movement and skin integrity were assessed every 10 min. All patients achieved complete symptom resolution within 20–50 min (median: 30 min) without complications. No cold‐induced skin injury occurred, and no additional treatment was required. While spontaneous recovery cannot be excluded, the relatively rapid improvement observed in this series suggests that direct ice packing may represent a safe, practical, and noninvasive management option for RFA‐related voice changes. Further studies are warranted to confirm its efficacy in larger and more diverse populations.

## 1. Introduction

Thermal ablation (TA), encompassing techniques such as radiofrequency ablation (RFA) and microwave ablation (MWA), has been demonstrated to be highly safe and effective for the treatment of both benign thyroid nodules [1, 2] and recurrent thyroid cancer [3, 4]. In patients undergoing RFA, the overall incidence of complications was reported to be 2.1% for benign thyroid nodules and 11.0% for recurrent thyroid cancer. Furthermore, the incidence of major complications was reported to be 1.3% for benign nodules and 6.7% for recurrent thyroid cancer [5]. Among these major complications, voice change resulting from nerve damage is the most common in various TA procedures [6, 7].

Although many cases of voice change following TA eventually resolve without intervention, the recovery process may take several weeks to months [7, 8], potentially affecting the patient’s quality of life during this time. Given this, timely and effective management of such symptoms is essential. Cold 5% dextrose in water (5% DW) injection has been proposed as a management strategy, with studies by Chung et al. [9] and Lee et al. [10] demonstrating its effectiveness in promoting symptom recovery. However, despite its clinical benefit, 5% DW injection may pose technical challenges, especially for less experienced operators unfamiliar with perithyroidal anatomy [11] and injection techniques. Therefore, a simpler and more broadly applicable alternative may provide clinical value, especially in settings where targeted perineural injection is difficult to perform promptly.

In this case series, we describe our clinical experience using direct ice packing as an alternative, noninvasive approach to manage voice changes encountered during or immediately after RFA. This technique was applied to a series of five patients at our institution who developed voice changes following RFA. Our aim is to share these cases to highlight the feasibility and practical value of direct ice packing as a simple and readily applicable strategy in clinical settings.

## 2. Materials and Methods

### 2.1. Patient Selection

This retrospective case series was approved by the Institutional Review Board of Chung Shan Medical University Hospital (IRB Number CS1‐23069). We reviewed medical records from January 2018 to January 2025 and identified five patients who developed voice changes during or immediately after RFA for benign thyroid nodules. The diagnosis of benign thyroid nodules was confirmed through at least two fine needle aspiration (FNA) procedures prior to ablation [12]. Collected data included patient demographics, nodule characteristics (size, volume, and location), onset of voice change symptoms, and the time interval to complete voice recovery, which was measured from the initiation of direct ice packing. Nodule volume was calculated using the formula *V* = π*abc*/6, where *V* represents the volume, *a* is the largest diameter, and *b* and *c* are the two perpendicular diameters [13].

### 2.2. TA Procedure

All RFA procedures were performed in an outpatient setting by a radiologist (KL Cheng) with approximately 7 years of experience. Procedures were conducted under real‐time ultrasound guidance using either 18‐ or 19‐gauge thyroid‐specific internally cooled electrodes (StarMed, Korea; RF Medical, Korea). Local anesthesia with 2% lidocaine was administered, and RFA was performed using the trans‐isthmic approach and moving‐shot technique under continuous ultrasound guidance [14]. Critical anatomical structures, including blood vessels and the recurrent laryngeal nerve (RLN), were carefully visualized or inferred and monitored to minimize risk. Hydrodissection using cold 5% DW was not routinely performed.

### 2.3. Ice Packing Protocol for Voice Change

Sterile gloves filled with ice cubes were routinely prepared prior to RFA (Figure 1) to ensure immediate availability. Voice quality was monitored during the procedure by intermittent self‐assessment [15]. If a patient‐reported voice changes during or shortly after the procedure, the procedure was halted, and laryngeal ultrasound was promptly performed to assess vocal cord (VC) mobility [16, 17]. Although the RLN is not directly visible on ultrasound, its anatomical location in the tracheoesophageal groove is known to correlate with thermal injury‐induced voice changes [5, 18].

Figure 1Sterile ice pack preparation and application during thyroid TA. Prior to initiating thermal ablation, (A) ice cubes (arrow) are inserted into a sterile glove. (B) Subsequently, the end of the glove is securely tied off (arrow) to form a sterile ice pack. (C) Once a voice change is detected during TA, the pre‐prepared sterile ice pack is applied directly to the skin surface at the site of the procedure for cooling.

**Figure:**
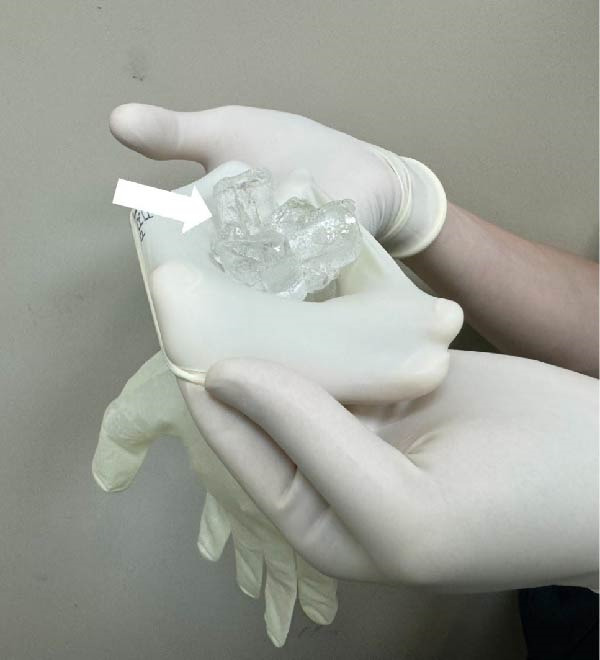
(A)

**Figure:**
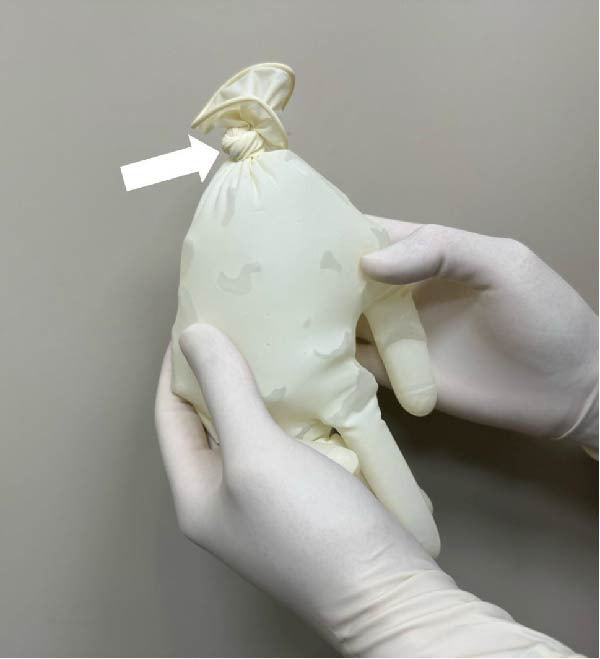
(B)

**Figure:**
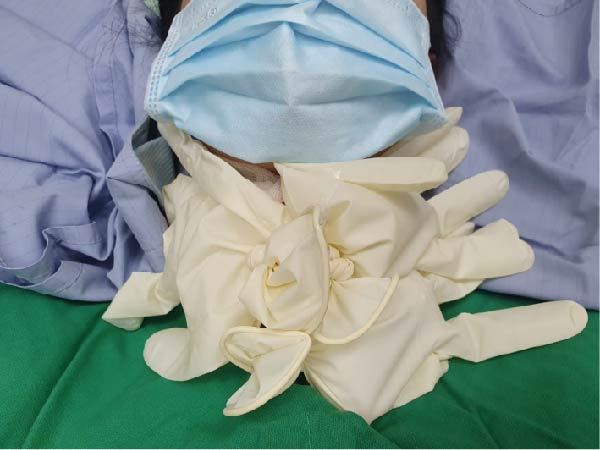
(C)

Upon detecting impaired VC movement or patient‐reported voice alteration, the ablation procedure was immediately halted, and the prepared ice glove was applied directly to the skin over the thyroid gland (Figure 1C). During ice application, laryngeal ultrasound was repeated every 10 min to monitor VC mobility, and the overlying skin was examined at the same intervals for signs of cold‐induced injury. The duration of ice application was recorded for each case. Patients were contacted by telephone the following day to confirm the resolution of symptoms.

## 3. Case Presentation

Table 1 summarizes the demographic and clinical characteristics of five patients (3 females and 2 males; age range: 44–67 years) who experienced voice changes during or immediately after RFA for benign thyroid nodules. The nodule volumes ranged from 1.02 to 10.62 mL, with maximal diameters ranging from 15.9 to 30.2 mm.

**Table 1:** Demographic and clinical characteristics of patients experiencing voice change following thermal ablation.

Patient number	Age (y/o)	Sex	Size (mm)	Volume (mL)	Location
1	54	F	11.0 × 18.5 × 22.9	2.44	Right
2	45	M	20.3 × 25.3 × 29.5	7.93	Left
3	44	M	17.9 × 16.7 × 24.5	3.83	Left
4	67	F	23.7 × 28.3 × 30.2	10.62	Left
5	53	F	9.3 × 13.1 × 15.9	1.02	Right

All RFA procedures were performed using 18‐ or 19‐gauge electrodes with active tips measuring 5–7 mm. Ablation durations ranged from 290 to 555 s, maximum power from 20 to 40 W, and total delivered energy from 0.41 to 2.70 kcal (Table 2).

**Table 2:** Detailed procedure parameters and voice change outcomes in thermal ablation patients.

Patient number	Needle size (Ga)	Active tip (mm)	Ablation time (s)	Max. power (watt)	Total energy (Kcal)	Symptom onset of voice change	Time interval until voice totally recovery^a^
1	18	5	418	20	1.40	During	20 min
2	18	7	494	40	2.37	During	20 min
3	19	5	319	20	0.41	During	30 min
4	18	7	555	35	2.70	During	30 min
5	18	5	290	20	0.70	Immediately after	50 min

^a^Direct ice packing was applied.

Voice changes occurred during ablation in four patients and immediately after the procedure in one patient (Table 2). Upon the onset of symptoms, laryngeal ultrasound revealed decreased mobility of the ipsilateral VC in all cases. Recovery times, calculated from the initiation of direct ice packing, ranged from 20 to 50 min, with a median duration of 30 min. None of the patients experienced cold‐induced skin injuries associated with the ice application. All patients achieved complete resolution of symptoms without requiring additional interventions.

## 4. Discussion

Voice change is a well‐recognized complication during RFA for thyroid nodules, often attributed to thermal injury to the RLN or its adjacent structures. In this case series, we described five patients who developed voice changes during or immediately after RFA and were managed using direct ice packing. All patients achieved full recovery within 20–50 min, with no additional treatment required. These preliminary observations suggest that immediate application of direct ice packing could serve as a simple, noninvasive, and potentially effective intervention for RFA‐induced voice changes in outpatient settings.

Although the present case series focused on the application of direct ice packing, prior literature has described cold 5% DW injection as an effective strategy for managing voice changes during thyroid RFA. Notably, when injected directly into the perithyroidal space adjacent to the injured nerve, cold 5% DW has been associated with rapid symptom relief, with most reported cases showing immediate improvement during or shortly after injection [9, 10, 19]. In contrast, the surface cooling method used in our cases likely results in a slower temperature decrease at the site of neural irritation. This difference in the mechanism of action may help explain the relatively longer recovery times observed in our patients. Nevertheless, all cases in our series achieved full symptom resolution without the need for additional intervention, suggesting that direct ice packing may serve as a simple and accessible alternative for immediate symptom management in clinical practice.

While this case series demonstrated that all patients experienced voice recovery within a relatively short timeframe after direct ice packing, the possibility of spontaneous resolution cannot be entirely ruled out. Most nerve injuries resulting from TA have been reported as temporary, with symptom resolution typically occurring within one to 3 months after the procedure [7, 8]. This suggests that even without any intervention, voice changes may eventually improve over time. In our cases, recovery occurred within 20–50 min, which appears considerably shorter than the usual recovery period described in the literature. Although this case series cannot confirm a causal relationship, the observed outcomes may support the potential benefit of immediate surface cooling in promoting faster symptom relief following suspected nerve irritation.

Although direct ice packing is simple and noninvasive, the risk of cold‐induced skin injury should not be overlooked. Previous reports have documented frostbite resulting from prolonged or improper ice application, especially in the absence of adequate monitoring [20, 21]. In our series, VC activity was assessed every 10 min using laryngeal ultrasound until symmetric mobility returned (Figure 2), and patients confirmed recovery in voice. At the same intervals, the overlying skin was examined for signs of cold‐induced injury. No patients developed frostbite or related complications. These findings suggest that with proper monitoring and controlled application, direct ice packing can be safely implemented during RFA.

Figure 2Laryngeal ultrasound findings following voice change and after ice packing. (A) Transverse laryngeal ultrasound shows poor visualization of the right false vocal cord (RFC, arrows), suggesting right vocal cord paralysis. (B) After direct ice packing treatment, the RFC becomes clearly visible, indicating recovery of vocal cord function. LFC, left false vocal cord; RFC, right false vocal cord.

**Figure:**
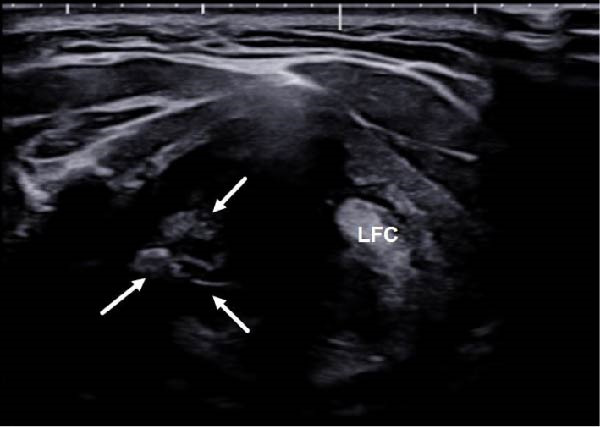
(A)

**Figure:**
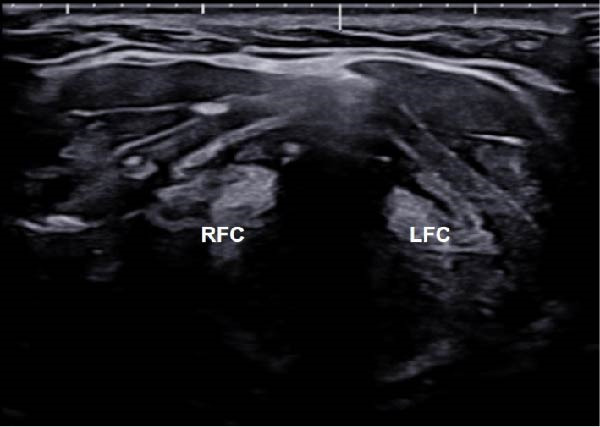
(B)

This case series has several limitations. First, the number of cases was small, and the retrospective design may introduce selection and information bias. Second, there was no direct comparison group, such as patients managed with a cold 5% DW injection, which limits our ability to evaluate relative efficacy. Third, all cases involved benign thyroid nodules; thus, the findings may not be generalizable to patients undergoing RFA for recurrent thyroid cancer, where the anatomy and risk of nerve injury may differ. Fourth, hydrodissection was not used during the procedures, which could potentially reduce the risk of nerve damage [8]. Finally, VC function was assessed using laryngeal ultrasound rather than laryngoscopy, while voice quality was monitored during the procedure by intermittent self‐assessment. Although laryngoscopy remains the most objective method for evaluating VC function, and intra‐procedural use has been explored in some studies [22, 23], its routine implementation may be limited by practical constraints. Prior studies have demonstrated that laryngeal ultrasound is a highly accurate tool that allows real‐time evaluation of VC movement during RFA [16]. Nonetheless, future research should aim to establish more objective and standardized protocols for intra‐procedural voice monitoring during TA.

## 5. Conclusion

In this case series, direct ice packing was used as a noninvasive management strategy for voice changes occurring during or immediately after RFA for benign thyroid nodules. All patients experienced full recovery within a brief timeframe and without observed complications. Although the possibility of spontaneous recovery cannot be excluded, the timely symptom resolution observed in our cases suggests that direct ice packing may offer a safe and practical option for outpatient management. Further prospective studies with larger sample sizes and comparative groups are warranted to validate its efficacy and broader clinical applicability.

## Funding

This study was sponsored by the Chung Shan Medical University Hospital Research Program (Grants CSH‐2024‐A‐024 and CSH‐2025‐A‐012), awarded to Dr. Kai‐Lun Cheng.

## Ethics Statement

This retrospective case series was approved by the Institutional Review Board of Chung Shan Medical University Hospital (IRB Number CS1‐23069). The study was conducted in accordance with the principles of the Declaration of Helsinki and the Good Clinical Practice (GCP) guidelines.

## Consent

Written informed consent was waived by the Institutional Review Board of Chung Shan Medical University Hospital due to the retrospective nature of the study.

## Conflicts of Interest

The authors declare no conflicts of interest.

## Data Availability

The data that support the findings of this study are available upon request from the corresponding author. The data are not publicly available due to privacy or ethical restrictions.
